# 4,4′-Bipyridinium bis­(oxalato-κ^2^ *O* ^1^,*O* ^2^)cuprate(II): an ion-pair complex

**DOI:** 10.1107/S1600536809039117

**Published:** 2009-10-03

**Authors:** Lai-Jun Zhang, Xing-Can Shen, Hong Liang

**Affiliations:** a Department of Chemistry, Shangrao Normal University, Shangrao 334001, People’s Republic of China; b School of Chemistry and Chemical Engineering, Nanjing University, Nanjing 210093, People’s Republic of China; c Key Laboratory of Medicinal Chemical Resources and Molecular Engineering, School of Chemistry and Chemical Engineering, Guangxi Normal University, Guilin 541004, People’s Republic of China

## Abstract

The title compound, (C_10_H_10_N_2_)[Cu(C_2_O_4_)_2_] or (4,4′-H_2_bpy)[Cu(ox)_2_] (bpy is 4,4′-bipyridine and ox is oxalate), is an ion-pair complex comprising a protonated 4,4′-bipyridinium dication and a square-planar dioxalatocopper(II) dianion. In the centrosymmetric dianion, the Cu^II^ centre is coordinated by four O atoms from the two dicrete oxalate ligands [Cu—O = 1.9245 (19) and 1.9252 (17) Å], while the planar dications are also centrosymmetric. Inter-species N—H⋯O hydrogen bonds link the cations and anions into one-dimensional chains and, together with weak intra-ion C—H⋯O inter­actions, give a two-dimensional sheet structure.

## Related literature

For related background, see: Ren *et al.* (2007 ▶). For related structures, see, for example: Bukowska-Strzyzewska & Tosik (1979 ▶); Crawford *et al.* (2004 ▶); Diallo *et al.* (2008 ▶); Dou *et al.* (2007 ▶); Madhu & Das (2004 ▶); Näther *et al.* (2001 ▶); Tosik *et al.* (1990 ▶); Willett *et al.* (2006 ▶).
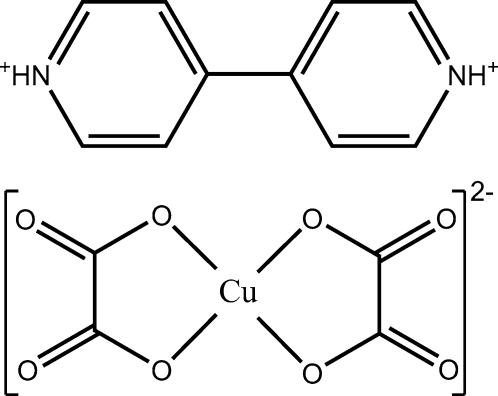


## Experimental

### 

#### Crystal data

(C_10_H_10_N_2_)[Cu(C_2_O_4_)_2_]
*M* *_r_* = 397.79
Triclinic, 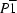

*a* = 3.6900 (7) Å
*b* = 9.950 (2) Å
*c* = 10.230 (2) Å
α = 113.77 (3)°
β = 98.43 (3)°
γ = 97.89 (3)°
*V* = 331.93 (15) Å^3^
*Z* = 1
Mo *K*α radiation
μ = 1.70 mm^−1^
*T* = 293 K
0.41 × 0.27 × 0.22 mm

#### Data collection

Bruker APEXII CCD area-detector diffractometer
Absorption correction: multi-scan (**SADABS**; Bruker, 2005 ▶) *T*_min_ = 0.527, *T*_max_ = 0.705
1761 measured reflections
1168 independent reflections
1126 reflections with *I* > 2σ(*I*)
*R*_int_ = 0.013

#### Refinement

*R*[*F* ^2^ > 2σ(*F* ^2^)] = 0.027
*wR*(*F* ^2^) = 0.073
*S* = 1.09
1168 reflections
115 parameters
H-atom parameters constrained
Δρ_max_ = 0.30 e Å^−3^
Δρ_min_ = −0.29 e Å^−3^

### 

Data collection: *APEX2* (Bruker, 2005 ▶); cell refinement: *SAINT-Plus* (Bruker, 2005 ▶); data reduction: *SAINT-Plus*; program(s) used to solve structure: *SHELXS97* (Sheldrick, 2008 ▶); program(s) used to refine structure: *SHELXL97* (Sheldrick, 2008 ▶); molecular graphics: *SHELXTL* (Sheldrick, 2008 ▶); software used to prepare material for publication: *SHELXTL*.

## Supplementary Material

Crystal structure: contains datablocks I, global. DOI: 10.1107/S1600536809039117/zs2010sup1.cif

Structure factors: contains datablocks I. DOI: 10.1107/S1600536809039117/zs2010Isup2.hkl

Additional supplementary materials:  crystallographic information; 3D view; checkCIF report

## Figures and Tables

**Table 1:** Hydrogen-bond geometry (Å, °)

*D*—H⋯*A*	*D*—H	H⋯*A*	*D*⋯*A*	*D*—H⋯*A*
N1—H1*A*⋯O4^i^	0.86	2.02	2.755 (3)	143
N1—H1*A*⋯O1^i^	0.86	2.21	2.880 (3)	135
C1—H1⋯O4^ii^	0.93	2.49	3.381 (3)	160
C2—H2⋯O3^ii^	0.93	2.57	3.195 (3)	125
C4—H4⋯O2	0.93	2.42	3.272 (3)	153
C5—H5⋯O1	0.93	2.46	3.215 (3)	138

Symmetry codes: (i) 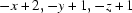; (ii) 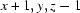.

**Fig. 1.**
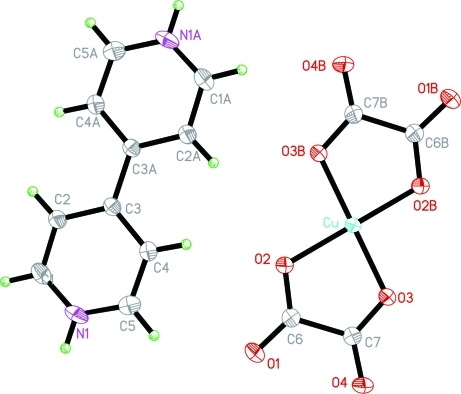
Molecular configuration and atom numbering scheme for the discrete dication and dianion species in (I). Displacement ellipsoids are drawn at the 50% probability level. Both cation and anion have crystallographic inversion symmetry. Symmetry codes: (A) -x + 1, -y, -z; (B) -x, -y, -z + 1.

**Fig. 2.**
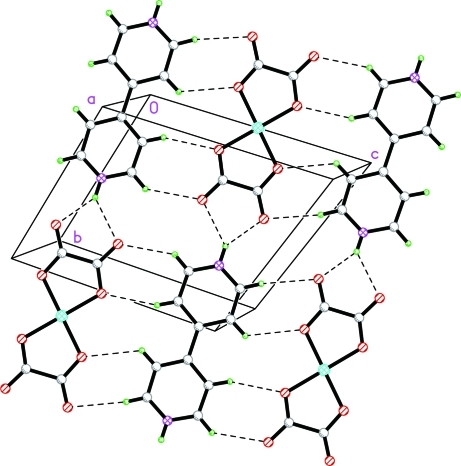
The two-dimensional hydrogen-bonded sheet structure of (I) showing intra- and intermolecular hydrogen bonds as dashed lines.

## supplementary crystallographic information

### Comment

Currently, *in situ* chemical reactions are of considerable interest, providing a powerful route for the preparation of novel crystals with unexpected structures, e.g. Ren *et al.* (2007) has reported the hydrothermal synthesis of a new coordination polymer [Cu(ox)(4,4'-bpy)]_n _(bpy = 4,4'-bipyridine; ox = oxalate^2-^) resulting from the decomposition of furan-2-carboxylic acid to give the ox^2-^ ligand in the reaction of this acid with Cu(NO_3_)_2_.3H_2_O and 4,4'-bpy. In the work reported here, a novel ion-pair complex, (4,4'-H_2_bpy)[Cu(ox)_2_] (I) was obtained by using sodium 2-hydroxyphosphonocarboxylate as the ox^2-^ source and CuCl_2_ . 2H_2_O as the copper source. Copper(II) is a relatively strong oxidant with the ability to oxidise the sodium 2-hydroxyphosphonocarboxylic giving ox^2-^ which is generated *in situ*, resulting in formation of the title compound, the structure of which is reported here.

The structure of the (I) shows an ion-pair complex comprising a protonated 4,4'-bipyridine dication, (4,4'-H_2_bpy)^2+^ (Bukowska-Strzyzewska *et al.*, 1979; Crawford *et al.*, 2004; Dou *et al.*, 2007; Madhu *et al.*, 2004; Näther *et al.*, 2001; Tosik *et al.*, 1990; Willett *et al.*, 2006), and a [Cu(C_2_O_4_)_2_]^2-^ anion (Fig. 1). The discrete bis-(oxalato)copper(II) dianions have square planar CuO_4_ stereochemistry, comprising four O donor atoms from two oxalate ligands [Cu–O, 1.9245 (19), 1.9252 (17) Å] and lie across inversion centres in the unit cell. In the axial sites, the Cu–O_oxalate_ contacts are 2.920 (2) Å. With the 4,4'-bipyridine dications, the pyridine rings are coplanar and also have crystallographic inversion symmetry. Interamolecular N—H···O hydrogen bonds (Table 1) link the cations and anions into one-dimensional chains and together with weak intramolecular C–H···O interactions give two-dimensional sheet structures which layer down the *a* direction in the unit cell (Fig. 2).

### Experimental

Sodium 2-hydroxyphosphonocarboxylate (1 mmol) was dissolved in 10 ml of 50% ethanol-water after which 1 mmol of copper(II) chloride dihydrate and 1 mmol of 4,4'-bipyridine were added in sequence. After stirring for 10 min, the resulting mixture was transferred into a Teflon-lined stainless steel vessel (15 ml) which was sealed and heated at 110 °C for four days, then allowed to cool to room temperature. The blue-green single crystal blocks of the title compound (I), together with yellow-red prismatic crystals of a second unidentified component were obtained. The yield of (I) was *ca.* 25% (based on copper). IR (cm^-1^, KBr): 3439, 3110, 3060, 2921, 1665, 1581, 1487, 1409, 1273, 1205 1106, 1000, 892, 827, 798.

### Refinement

All H atoms were geometrically placed and refined using a riding model approximation, with C—H = 0.93 Å or N—H = 0.86 Å and *U*_iso_(H) = 1.2*U*_eq_ (C, N).

### Crystal data

**Table:**

(C_10_H_10_N_2_)[Cu(C_2_O_4_)_2_]	*Z* = 1
*M**_r_* = 397.79	*F*(000) = 201
Triclinic, *P*1	*D*_x_ = 1.990 Mg m^−^^3^
Hall symbol: -P 1	Mo *K*α radiation, λ = 0.71073 Å
*a* = 3.6900 (7) Å	Cell parameters from 1168 reflections
*b* = 9.950 (2) Å	θ = 2.2–25.1°
*c* = 10.230 (2) Å	µ = 1.70 mm^−^^1^
α = 113.77 (3)°	*T* = 293 K
β = 98.43 (3)°	Block, green-blue
γ = 97.89 (3)°	0.41 × 0.27 × 0.22 mm
*V* = 331.93 (15) Å^3^	

### Data collection

**Table:**

Bruker APEXII CCD area-detector diffractometer	1168 independent reflections
Radiation source: fine-focus sealed tube	1126 reflections with *I* > 2σ(*I*)
graphite	*R*_int_ = 0.013
φ and ω scans	θ_max_ = 25.1°, θ_min_ = 2.2°
Absorption correction: multi-scan (*SADABS*; Bruker, 2005)	*h* = −4→4
*T*_min_ = 0.527, *T*_max_ = 0.705	*k* = −9→11
1761 measured reflections	*l* = −11→12

### Refinement

**Table:**

Refinement on *F*^2^	Primary atom site location: structure-invariant direct methods
Least-squares matrix: full	Secondary atom site location: difference Fourier map
*R*[*F*^2^ > 2σ(*F*^2^)] = 0.027	Hydrogen site location: inferred from neighbouring sites
*wR*(*F*^2^) = 0.073	H-atom parameters constrained
*S* = 1.09	*w* = 1/[σ^2^(*F*_o_^2^) + (0.0434*P*)^2^ + 0.1814*P*] where *P* = (*F*_o_^2^ + 2*F*_c_^2^)/3
1168 reflections	(Δ/σ)_max_ < 0.001
115 parameters	Δρ_max_ = 0.30 e Å^−^^3^
0 restraints	Δρ_min_ = −0.29 e Å^−^^3^

### Special details

**Table:**

Geometry. All e.s.d.'s (except the e.s.d. in the dihedral angle between two l.s. planes) are estimated using the full covariance matrix. The cell e.s.d.'s are taken into account individually in the estimation of e.s.d.'s in distances, angles and torsion angles; correlations between e.s.d.'s in cell parameters are only used when they are defined by crystal symmetry. An approximate (isotropic) treatment of cell e.s.d.'s is used for estimating e.s.d.'s involving l.s. planes.
Refinement. Refinement of *F*^2^ against ALL reflections. The weighted *R*-factor *wR* and goodness of fit *S* are based on *F*^2^, conventional *R*-factors *R* are based on *F*, with *F* set to zero for negative *F*^2^. The threshold expression of *F*^2^ > σ(*F*^2^) is used only for calculating *R*-factors(gt) *etc*. and is not relevant to the choice of reflections for refinement. *R*-factors based on *F*^2^ are statistically about twice as large as those based on *F*, and *R*- factors based on ALL data will be even larger.

### Fractional atomic coordinates and isotropic or equivalent isotropic displacement parameters (Å^2^)

**Table:**

	*x*	*y*	*z*	*U*_iso_*/*U*_eq_	
Cu1	0.0000	0.0000	0.5000	0.02273 (16)	
C1	0.9693 (6)	0.2924 (3)	0.0264 (3)	0.0263 (5)	
H1	1.0658	0.3392	−0.0273	0.032*	
C2	0.7599 (6)	0.1481 (3)	−0.0443 (3)	0.0228 (5)	
H2	0.7140	0.0971	−0.1459	0.027*	
C3	0.6154 (6)	0.0779 (2)	0.0367 (2)	0.0182 (4)	
C4	0.6955 (7)	0.1599 (3)	0.1887 (3)	0.0265 (5)	
H4	0.6054	0.1165	0.2461	0.032*	
C5	0.9057 (7)	0.3037 (3)	0.2534 (3)	0.0312 (6)	
H5	0.9580	0.3582	0.3547	0.037*	
C6	0.4733 (6)	0.2586 (3)	0.5616 (3)	0.0232 (5)	
C7	0.3414 (6)	0.2770 (3)	0.7045 (2)	0.0224 (5)	
N1	1.0353 (5)	0.3657 (2)	0.1713 (2)	0.0273 (4)	
H1A	1.1664	0.4564	0.2135	0.033*	
O1	0.6801 (5)	0.3636 (2)	0.5609 (2)	0.0342 (4)	
O2	0.3514 (5)	0.12699 (19)	0.45407 (17)	0.0286 (4)	
O3	0.1187 (5)	0.16185 (19)	0.69418 (18)	0.0265 (4)	
O4	0.4569 (5)	0.39568 (19)	0.81441 (18)	0.0313 (4)	

### Atomic displacement parameters (Å^2^)

**Table:**

	*U*^11^	*U*^22^	*U*^33^	*U*^12^	*U*^13^	*U*^23^
Cu1	0.0285 (2)	0.0171 (2)	0.0166 (2)	−0.00496 (16)	0.00626 (16)	0.00411 (17)
C1	0.0250 (12)	0.0251 (12)	0.0318 (13)	0.0015 (10)	0.0060 (10)	0.0167 (11)
C2	0.0249 (11)	0.0219 (12)	0.0211 (11)	0.0009 (9)	0.0046 (9)	0.0102 (10)
C3	0.0155 (10)	0.0184 (11)	0.0216 (11)	0.0041 (9)	0.0043 (8)	0.0095 (9)
C4	0.0319 (13)	0.0245 (12)	0.0218 (12)	−0.0003 (10)	0.0082 (10)	0.0101 (10)
C5	0.0360 (14)	0.0262 (13)	0.0237 (13)	−0.0002 (11)	0.0047 (10)	0.0059 (11)
C6	0.0244 (11)	0.0224 (12)	0.0214 (12)	0.0018 (9)	0.0051 (9)	0.0092 (10)
C7	0.0235 (11)	0.0221 (12)	0.0208 (12)	0.0022 (9)	0.0046 (9)	0.0096 (10)
N1	0.0242 (10)	0.0160 (10)	0.0371 (12)	−0.0022 (8)	0.0040 (9)	0.0099 (9)
O1	0.0425 (10)	0.0237 (9)	0.0309 (10)	−0.0083 (8)	0.0111 (8)	0.0101 (8)
O2	0.0381 (9)	0.0205 (8)	0.0196 (8)	−0.0057 (7)	0.0102 (7)	0.0037 (7)
O3	0.0332 (9)	0.0208 (8)	0.0198 (8)	−0.0059 (7)	0.0079 (7)	0.0062 (7)
O4	0.0389 (10)	0.0203 (9)	0.0227 (9)	−0.0070 (7)	0.0063 (7)	0.0018 (7)

### Geometric parameters (Å, °)

**Table:**

Cu1—O3	1.9245 (19)	C4—C5	1.367 (4)
Cu1—O3^i^	1.9245 (19)	C4—H4	0.9300
Cu1—O2^i^	1.9252 (17)	C5—N1	1.331 (3)
Cu1—O2	1.9252 (17)	C5—H5	0.9300
C1—N1	1.327 (3)	C6—O1	1.210 (3)
C1—C2	1.369 (3)	C6—O2	1.288 (3)
C1—H1	0.9300	C6—C7	1.557 (3)
C2—C3	1.397 (3)	C7—O4	1.221 (3)
C2—H2	0.9300	C7—O3	1.272 (3)
C3—C4	1.395 (3)	N1—H1A	0.8600
C3—C3^ii^	1.485 (4)		
			
O3—Cu1—O3^i^	180.0	C5—C4—H4	119.9
O3—Cu1—O2^i^	93.91 (7)	C3—C4—H4	119.9
O3^i^—Cu1—O2^i^	86.09 (7)	N1—C5—C4	119.9 (2)
O3—Cu1—O2	86.09 (7)	N1—C5—H5	120.1
O3^i^—Cu1—O2	93.91 (7)	C4—C5—H5	120.1
O2^i^—Cu1—O2	180.00 (8)	O1—C6—O2	126.2 (2)
N1—C1—C2	120.4 (2)	O1—C6—C7	119.2 (2)
N1—C1—H1	119.8	O2—C6—C7	114.60 (19)
C2—C1—H1	119.8	O4—C7—O3	125.9 (2)
C1—C2—C3	119.7 (2)	O4—C7—C6	119.3 (2)
C1—C2—H2	120.1	O3—C7—C6	114.8 (2)
C3—C2—H2	120.1	C1—N1—C5	122.3 (2)
C4—C3—C2	117.5 (2)	C1—N1—H1A	118.9
C4—C3—C3^ii^	121.4 (2)	C5—N1—H1A	118.9
C2—C3—C3^ii^	121.1 (3)	C6—O2—Cu1	111.74 (14)
C5—C4—C3	120.3 (2)	C7—O3—Cu1	112.38 (14)

Symmetry codes: (i) −*x*, −*y*, −*z*+1; (ii) −*x*+1, −*y*, −*z*.

### Hydrogen-bond geometry (Å, °)

**Table:**

*D*—H···*A*	*D*—H	H···*A*	*D*···*A*	*D*—H···*A*
N1—H1A···O4^iii^	0.86	2.02	2.755 (3)	143
N1—H1A···O1^iii^	0.86	2.21	2.880 (3)	135
C1—H1···O4^iv^	0.93	2.49	3.381 (3)	160
C2—H2···O3^iv^	0.93	2.57	3.195 (3)	125
C4—H4···O2	0.93	2.42	3.272 (3)	153
C5—H5···O1	0.93	2.46	3.215 (3)	138

Symmetry codes: (iii) −*x*+2, −*y*+1, −*z*+1; (iv) *x*+1, *y*, *z*−1.
